# Editorial for the Special Issue “Current Development of Pediatric Minimally Invasive Surgery” of the Journal *Children*

**DOI:** 10.3390/children10101650

**Published:** 2023-10-03

**Authors:** Robert Bergholz, Thomas Franz Krebs

**Affiliations:** 1 Department of General, Visceral, Thoracic, Transplant and Pediatric Surgery, University Hospital of Schleswig-Holstein Campus Kiel, Arnold-Heller-Straße 3, 24105 Kiel, Germany; 2 Clinic for Pediatric Surgery, Childrens Hospital of Eastern Switzerland, 9006 Saint Gallen, Switzerland; thomas.krebs@kispisg.ch

Dear colleagues,

Pediatric minimally invasive surgery (MIS) epitomizes a dynamic and continuously evolving field that encompasses a broad range of procedures. From fetoscopic interventions in the prenatal population to robotic-assisted bariatric surgeries in overweight adolescents, the spectrum of techniques and patients encountered in pediatric MIS reflects its inherent intricacy and the fascination it inspires.

In the context of this particular Special Issue, our primary objective revolved around an extensive exploration of the expansive landscape of pediatric MIS. This comprehensive investigation encompassed a wide range of relevant aspects, including well-established procedures, pioneering and audacious concepts—even those yielding unfavorable outcomes, breakthrough technological advancements, potential hazards and challenges, contentious procedures, and invaluable insights drawn from our counterparts in the realms of general, thoracic, and visceral surgery.

Our intention was to transcend a simple retrospective appraisal by eschewing the common narrative of “what has been accomplished thus far?”. Instead, our aim was to assemble a compendium of reports from pioneering colleagues from diverse medical disciplines. These individuals have forged innovatively ahead, propelling developments in minimally invasive surgical interventions in order to enhance the welfare of pediatric patients.

We are pleased to say that our endeavors have borne fruit, and we believe that we have achieved our objective.

To improve the wellbeing of pediatric patients even before their birth, Thomas Kohl delves into the study of fetal surgery, offering insights into both established and emerging minimally invasive approaches. Expanding upon this, Susanne Eva Brunner provides an exhaustive review comparing bimanual techniques and procedures in fetal surgery with single-instrument interventions. Additionally, Lidya-Olgu Durmaz sheds light on the use of minimally invasive techniques in fetal surgery for the treatment of gastroschisis.

Regarding infants and congenital malformations, Anne-Sophie Holler describes the endoscopic magnetic-assisted anastomosing technique, specifically designed for the repair of esophageal atresia. Additionally, Julia Küppers presents an innovative approach termed “endoscopic percutaneous rectal anoplasty”, which has garnered considerable attention from the esteemed Alberto Pena, a prominent authority on anorectal malformation repair, prompting subsequent scholarly discourse. In a closely related context, Ulrike Metzger provides valuable insights into a recently modified approach involving transanal endoscopic-assisted pull-through colectomy targeted toward pediatric patients afflicted by high intestinal aganglionosis. These developments in pediatric surgical interventions offer promising avenues for improved outcomes and enhanced patient care.

When it comes to general pediatric surgical procedures, this comprehensive compilation includes many notable contributions. Hanna Noemi Stundner Ladenhauf presents a meticulous evaluation of the minimally invasive approach in managing traumatic pancreatic ruptures, shedding light on its efficacy and potential benefits. Armin-Johannes Michel provides a detailed account of the thoracoscope-guided pericostal suture technique, showcasing its efficacy as a robust fixation method for closing congenital diaphragmatic hernias. The state-of-the-art of advancements in the field of minimally invasive repair of pectus excavatum (MIRPE) is expertly detailed by Frank-Martin Haecker, as he reveals the current developments and techniques employed in this realm. In a similar vein, Christian Tomuschat delves into the boundaries and limitations encountered in laparoscopic partial splenectomy, providing valuable insights into the challenges associated with this minimally invasive procedure.

The domain of pediatric surgery encompasses not only general procedures but also extends to minimally invasive urological interventions. In this context, Frank-Martin Haecker contributes valuable insights by reporting on the combined antegrade and retrograde endoscopic injection technique into the bladder neck employed in children with neurogenic bladder disorders. Furthermore, Christian Kruppa presents a fascinating approach to vesicoscopic cross-trigonal ureteral reimplantation, specifically addressing vesicoureteral reflux. This innovative technique offers a compelling avenue for the treatment and resolution of this condition. These advancements in minimally invasive urological procedures within pediatric surgery hold great promise for enhanced patient outcomes and an improved quality of care.

Mareike Grosshauser contributes an insightful examination of interdisciplinary knowledge transfer within the study of minimally invasive pediatric surgery. Similarly, Tatjana Tamara König delves into the topic of telementoring in the context of minimally invasive esophageal atresia repair, shedding light on the potential benefits and implications of this approach. Expanding our understanding, Andrea Schmedding presents a comprehensive nationwide assessment of the current landscape surrounding laparoscopic pediatric inguinal hernia surgery, drawing upon an extensive dataset. Furthermore, Małgorzata Sobol offers a unique perspective by focusing on the parental experience of minimally invasive surgery, specifically examining the role of parents’ time perspective as a predictive factor for their child’s postsurgical pain and emergence delirium, and the potential manifestation of post-traumatic stress disorder in parents.

The integration of robotic-assisted surgery will be pivotal element in the future landscape of minimally invasive pediatric surgical procedures. Ewan Brownlee and Mark Slack delve into this paradigm-shifting subject, offering an insightful report on the novel Versius surgical robotic system developed by Cambridge Medical (CMR), exploring its potential utility within the pediatric population. In a related investigation, Marit Kayser presents an evaluation of the Versius system for pediatric surgery, employing inanimate models that simulate small infants and, thus, providing valuable insights into its feasibility and effectiveness. Within the pages of this publication, we also encounter a groundbreaking contribution by Jürgen Holzer, who provide the world’s inaugural account of a pediatric pyeloplasty performed using the Senhance® robotic system. This pioneering achievement marks a significant milestone in the application of robotic technology in the pediatric surgical domain. Additionally, Elisabeth Ammer explores the phenomenon of “robotic anxiety” and elucidate parents’ perception of robot-assisted pediatric surgery and its impact on their children. This investigation sheds light on the psychological aspects and human factors associated with the adoption of robotic systems in pediatric surgical contexts. Finally, Thomas Krebs offers a comprehensive review encompassing diverse aspects of robotic-assisted pediatric surgery. Our collective insights and synthesis of existing knowledge serve to enrich our understanding and foster advancements in this rapidly evolving field.

Nevertheless, the possibilities of minimally invasive pediatric surgery transcend the traditional boundaries of abdominal and thoracic procedures. Within the field of pediatric orthopedics and orthopedic trauma, Karsten Krohn unveils a pioneering technique involving the dual pre-bending of an intramedullary nail to treat diametaphyseal fractures of the distal radius in children. This innovative approach not only renders the procedure and osteosynthesis less invasive but also holds promise for improved patient outcomes. Moreover, the use of resorbable implants presents an intriguing possibility for circumventing the need for subsequent explantation surgeries; this is an area explored by Christoph Roeder. Through a pilot study, he investigates the application of biodegradable intramedullary nailing, specifically for forearm fractures in the pediatric population. His findings shed light on the feasibility and potential benefits of this approach, paving the way for future advancements in pediatric orthopedic interventions. Similarly, Pascal Heye contributes to this line of inquiry by delving into the utilization of resorbable implants for osteosynthesis in pediatric cases. His research further expands our understanding of this evolving field, exploring possibilities for enhanced treatment modalities and improved patient outcomes. Furthermore, Mathis Wegner presents his insights into a minimally invasive orthosis designed to address a fixed equinus deformity following a modified transverse Vulpius procedure. This novel orthosis holds promise in rectifying this deformity while minimizing invasiveness, thus offering potential benefits to pediatric patients.

These contributions to the knowledge of minimally invasive pediatric surgery collectively underscore the constant pursuit of innovative techniques and advancements aimed at optimizing patient care and improving long-term outcomes.

This remarkable collection merits great attention, as it encompasses 27 pioneering works by surgeons who fearlessly expand the horizons of minimally invasive pediatric surgery.

We thank all the contributors, and congratulate them on their excellent work.

Best regards from Sankt Gallen, Switzerland and Kiel, Germany.

Thomas Franz Krebs and Robert Bergholz.

